# The Reconstruction of the Nasal Columella Defect Using Domino Flaps

**DOI:** 10.1055/a-2309-1701

**Published:** 2024-06-19

**Authors:** Le Diep Linh, Luu Phuong Lan, Nguyen Phuong Tien, Nguyen Quoc Manh, Vu Ngoc Lam, Nguyen Quang Duc

**Affiliations:** 1 Center for Craniofacial and Plastic Surgery, 108 Military Central Hospital, Hanoi, Vietnam; 2 Department of Plastic and Reconstructive Surgery, Hanoi Medical University, Hanoi, Vietnam

**Keywords:** nasal columella defect, nasocheek island flap, horizontal upper lip island flap, Domino flaps

## Abstract

Skin defects of the total nasal columella can significantly impact both nasal respiratory function and aesthetics. The reconstruction of total columella is a complex process and represents a significant challenge for plastic surgeons. Various factors can cause the loss of the columella. Numerous columella reconstruction procedures have been proposed, each with their own set of advantages and disadvantages. The main issues to address include the need for regional flaps from the forehead or nasofacial sulcus, a long pedicle to reach the columella, and the double angular folding that causes a risk of malnutrition or venous congestion. Additionally, using horizontal nasolabial flaps may lead to deformation of the upper lip.

In this study, we present a new procedure to reconstruct the nasal columella using “Domino flaps” with two flaps (the horizontal upper lip island flap and nasocheek island flap). This new procedure ensures adequate skin for reconstruction of nasal columella and partial tip, minimizes rotation angle, reduces the angular folding of the pedicle, furthermore limits deformation of the upper lip. “Domino flaps” are a valuable option for surgeons when reconstructing the total nasal columella. However, it is important to consider whether the patient has a beard at the donor sites.

## Introduction

The nasal columella is a nasal subunit that determines the projection of the tip and the connection between the nose and lip. There are a wide range of medical conditions causing columella defects, which are trauma, tumor resection, vascular malformations and congenital agenesis, ischemic lesions, infections, or aesthetic surgery complications. It is crucial to reconstruct the nasal columella carefully to maintain both the functionality of the nasal respiratory system and aesthetic appearance. However, the reconstruction of total columella is no simple task, even though it is just one small subunit of the nose. The challenge arises because of a scarcity of adjacent tissue available for reconstruction, as well as the columella's distinctive contour and discrete border. To cover the columella, skin can be taken from the nasal vestibule, lips, cheeks, forehead, or even from a distance. 1 Auricular chondrocutaneous composite graft or buccal mucosal flap can efficiently regenerate the columella. However, this may not perfectly match the original skin color of the nose. 2 3 The subnasale flap poses a size constraint, making it better suited for partial defects rather than complete ones. 4 The horizontal upper lip flap or vertical upper lip flap, can be utilized for the reconstruction of the columella. However, it is worth noting that this may lead to upper lip deformity or affect the contours of the philtrum. 5 Nasocheek flaps, nasolabial flaps, and forehead flaps have been utilized in numerous reports to reconstruct the entire columella defect. Initially, tubed pedicle flap was used. In the second stage, the pedicle was divided and transformed into a subcutaneous island pedicle flap, and passed under a long tunnel to the columella. Currently, the axial island flap has been improved to ensure blood supply. When the flap site is further from the columella, it requires dissection of the long pedicle and an increase in angulation due to the nose's central and protruding position on the face, posing a risk of obstruction in the pedicle. 1 6 7 8 9 10

We hereby present a straightforward procedure that uses “Domino flaps,” paired with the horizontal island upper lip flap and nasocheek island flap, to rebuild the entire skin of the nasal columella. This procedure is beneficial because it minimizes any negative effects at the donor site, provides a flap of ample size to reproduce the entire nasal columella, and ensures a similar color match.

## Idea

**Fig. 1:**
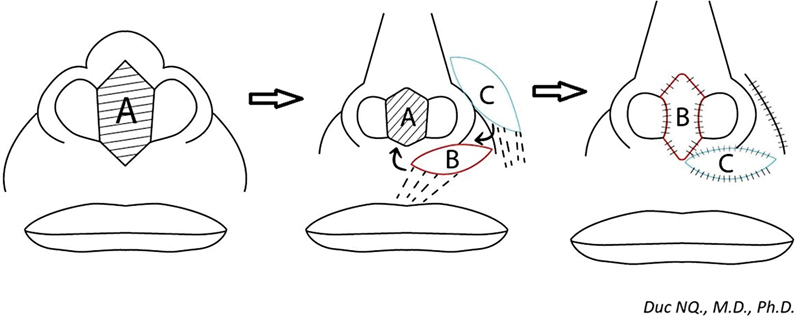
Drawing of surgical design. ( **A** ) Columella defect. ( **B** ) First flap—the horizontal upper lip island flap. ( **C** ) Second flap—the nasocheek island flap.

For the reconstruction of total columella, surgeons commonly use a forehead flap, nasolabial flaps, or nasocheek flaps to match the color and prevent distortion at the donor site. However, since these flaps are harvested from the areas distant from the columella, it often requires a two-stage operation or the use of an island flap with a long tunnel. These procedures mean the patient undergoes two surgeries or brings an increased risk of failure due to compression of the flap pedicle within the tunnel. To address these issues, we propose using “Domino flaps” composed of two flaps. The horizontal island upper lip flap rotates up to create a columella shape and the nasocheek island flap rotates inward to cover the donor site of the horizontal upper lip flap ( Fig. 1 ).

## Surgical Steps

### Under General Anesthesia

**Fig. 2:**
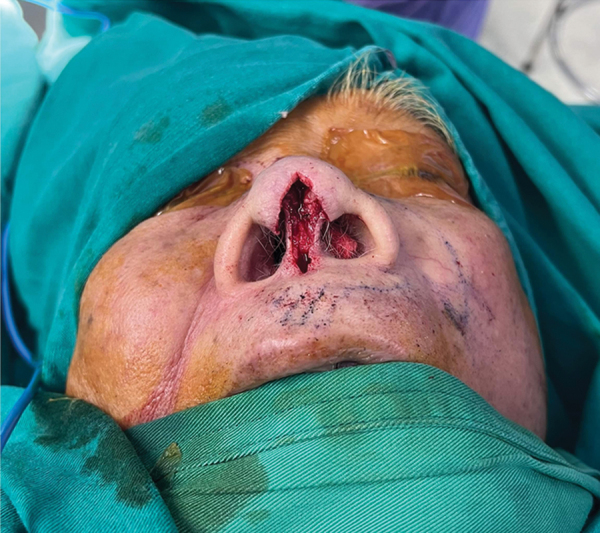
Impairment of the nasal columella structure.

**Fig. 3:**
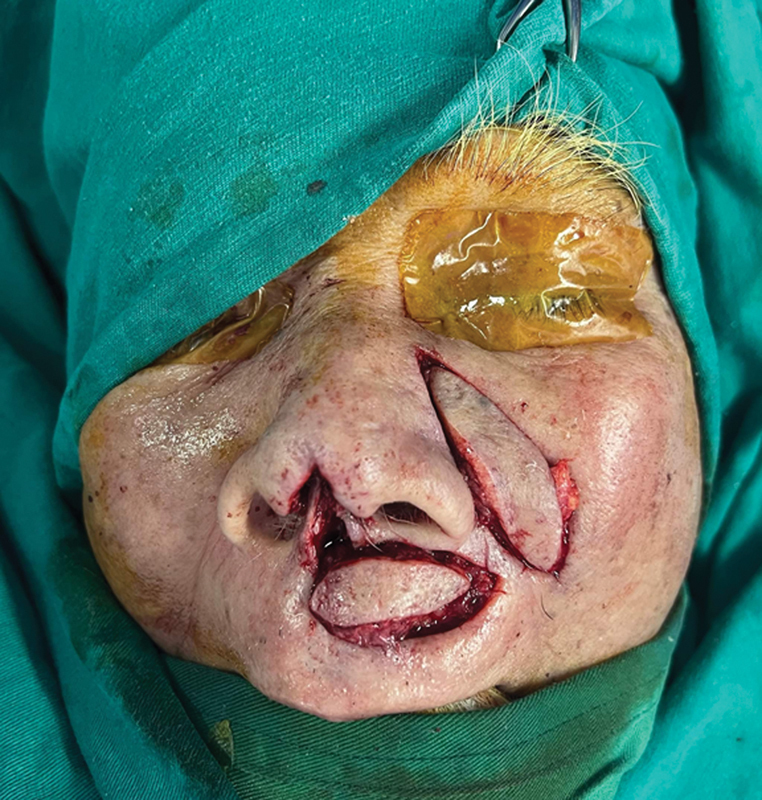
The horizontal upper lip island flap and nasocheek island flap.

**Fig. 4:**
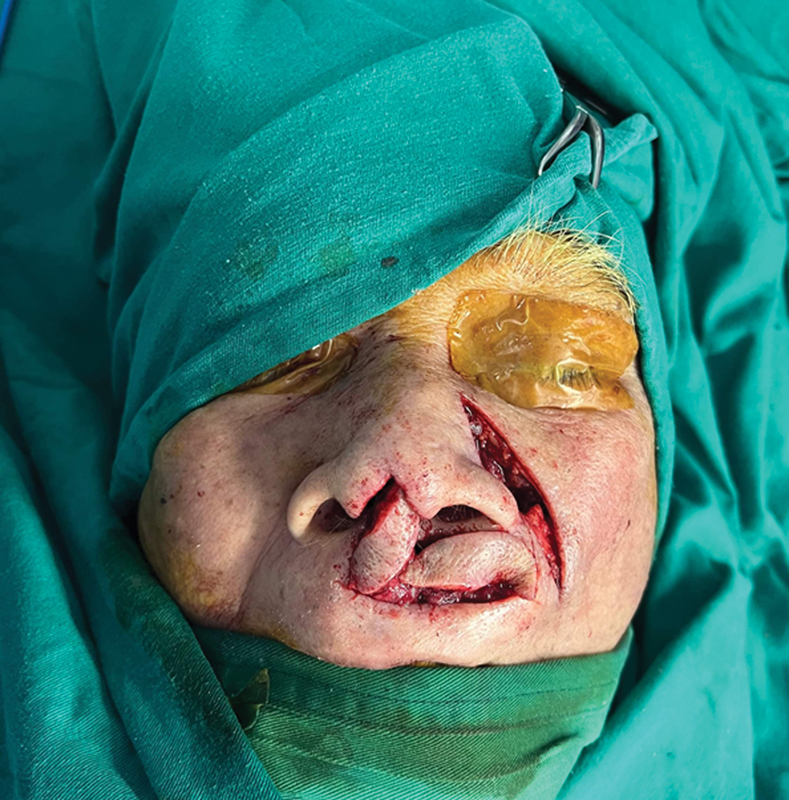
The flaps are adjusted, one at a time, similar to a Domino effect. The horizontal upper lip island flap is used to reconstruct the columella, while the nasocheek island flap covers the donor site of horizontal upper lip island flap.

**Fig. 5:**
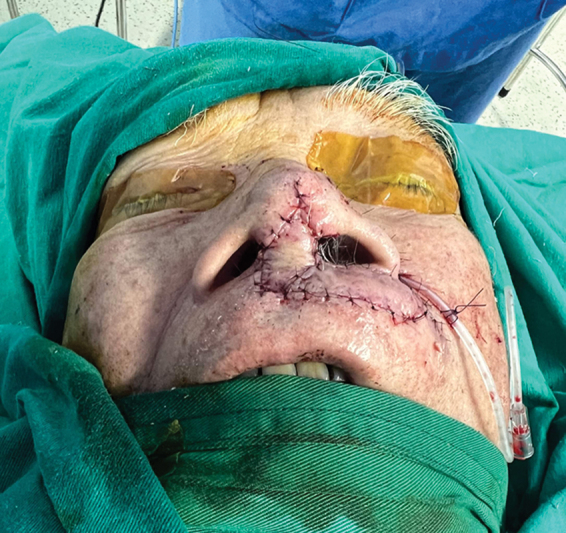
The flap is secured into the designated place.

1. The length and width of the columella defect are determined ( Fig. 2 ).
2. Flap design: The first flap: This flap's axis runs horizontal to the upper lip, and its width matches the width of the columella. The length of the flap equals the width of the columella plus the length of the columella to be reproduced. The flap's pivot point is at the philtrum. The second flap is located on the same side as the first island flap. The axis of the flap is located in the nasofacial sulcus and the pivot point is at the intersection of the first and second flap axis. The dimensions of the flap are determined based on the size of the defect remaining after the first flap is taken.
3. The first flap (the horizontal upper lip island flap) surgery: Make an incision in the skin along the designed line. Next, dissect the myocutaneous island flap. The flap's pedicle contains the upper part of the orbicularis oculi muscle. Finally, rotate the flap 90 degrees to cover the columellar defect.
4. The second flap (the nasocheek island flap) surgery: dissect the skin flap with the central or lateral subcutaneous pedicle ( Fig. 3 ). Rotate the flap to cover the donor site's the first flap. ( Fig. 4 ).
5. The incision is closed using 5.0 vicryl sutures and 6.0 nylon sutures ( Fig. 5 ).
  Remove the sutures 7 days postsurgery.

## Discussion

Nowicki et al in 2020, provided a comprehensive overview of the current nasal columella reconstruction procedures. His statistical research was completed using PubMed, MEDLINE, and Cochrane Database, from 1947 to 2018, and obtained relevant 655 papers, 71 articles. 1 A variety of procedures for the columella reconstruction are discussed, including skin grafts, local flaps, regional flaps on the cheeks, lips, forehead, and free flaps. Each procedure has its own advantages and disadvantages.

Full-thickness skin grafts or auricular chondrocutaneous composite grafts are simple techniques. However, the graft size is small and tends to shrink and shows hyperpigmentation. They are appropriate for reconstructing total columella defects in children. 1 2 Local facial flaps for columella reconstruction were first presented by Blair and Byar in 1946. 14 These flaps are commonly named after their donor site. 1 The use of nasal vestibular flaps, including internal nasal vestibular flaps, subnasale flaps, and alar rim flaps are simple and effective for immediate reconstruction of the columella. Typically, they involve raising bilateral flaps with random blood supply, small in size, and the possibility of nasal hair present on the far side of the flap. These procedures are usually best suited for partial columella defects or short nasal columella. 4

Pincus and Bukachevsky in 1990, used two medial-based horizontal nasolabial flaps or horizontal upper lip flaps. The donor sites were close directly and caused the upper lip lift. This procedure is especially suitable for a short nasal columella. 11 The island philtrum flap, or paraphiltrum flaps with a large rotation angle, were also used to reconstruct the columella. However, the flap size was limited and it deformed the upper lip. 5 The buccal mucosa flap's color is less similar to the skin of the nose, so it is rarely used. 3

Nasolabial or melolabial flap, nasocheek or nasofacial flap, are typically used for reconstructing columella defects that are larger than 20 mm. They can be obtained in various sizes, and either one or both sides can be used simultaneously. These flaps are particularly useful for reconstruction of total columella, especially when there are accompanying nasal sidewall and vestibule defects. 1 6 9 10 The forehead flap is a fitting choice for rebuilding the columella, particularly when there is also a defect at the nasal tip or the ala nasi. 7 8 These flaps are used in a delayed, second-stage pedicle resection to ensure safety. However, their bulkiness can cause inconvenience and extend treatment time. To improve this, an island flap with a subcutaneous pedicle or vascular axis, such as a reverse lateral nasal artery pedicled nasolabial island flap, can be used. This requires only one surgery and involves creating a tunnel under the skin wide enough for the pedicle to pass. The flap is then brought to the columella, a location that effectively hides scars. However, if the flap is far from the defect, a relatively long pedicle needs to be dissected and bent twice. This can occasionally cause blockages, leading to venous congestion or malnutrition. 1 7 8 9 10 Free flaps such as the auricular helix free flap are not the first choice, but a viable option when local flaps or regional flaps are not suitable; however, they require high technical skills and have a high risk of failure. 1 12 Choosing a local or regional facial flap is usually primary option. The choice of procedure is determined by factors such as the size of the defect, its features, and the characteristics of the surrounding area.

“Domino flaps” were introduced by Wei et al in 2016 for the reconstruction of nasal columella deformities in patients with bilateral cleft lip sequelae scars. This procedure involved a group of flaps. The first flap was used to correct the nasal columella deformity by pushing from the central philtrum upwards. The second flap was an Abbe flap from the lower lip, rotated up to reconstruct the central philtrum. The author named these “Domino flaps” due to their sequential movement resembling falling dominoes. 13 This “Domino flaps” was appropriate for patients with bilateral cleft lip sequelae, as it could rectify nasal columella deformity and lip hypoplasia. However, a drawback is the inadequacy of the central philtrum flap to reach the tip in cases where the patient has a defect in the entire skin of the nose. Additionally, a patient needed to undergo two surgeries. After 3 weeks, the pedicle of the second flap was divided.

**Fig. 6:**
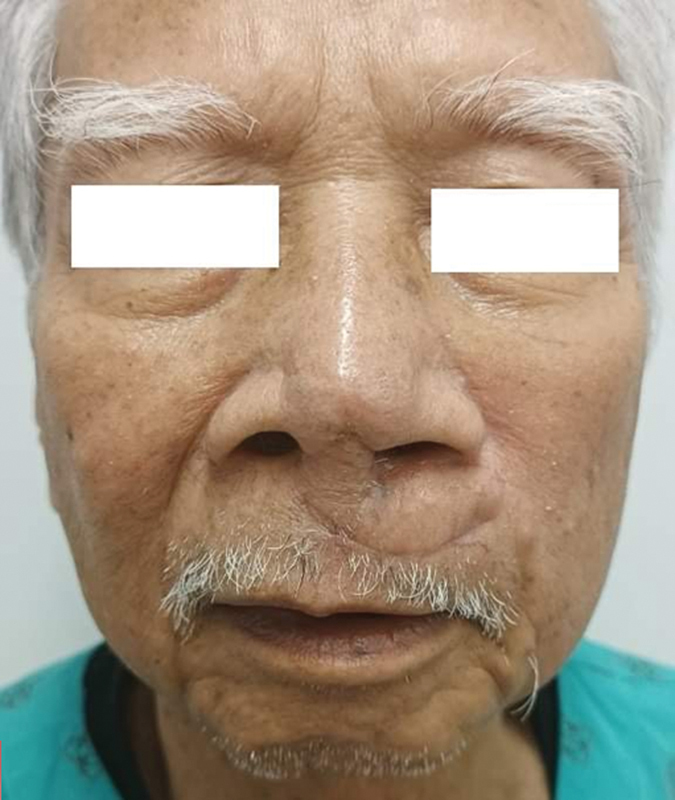
Three months after surgery.

To address these challenges, we have used the columella–upper lip–cheek “Domino flaps,” which consists of two flaps: the horizontal upper lip island flap and the nasocheek island flap. The horizontal island upper lip flap is an excellent choice for reconstruction of the total nasal columella, and part of the tip, given its large size and suitable color. The nasolabial flap can also be used instead of the nasocheek flap to cover donor site of the first flap. The donor site of the second flap can be easily directly closed. It also allows to maintain the philtrum and to avoid any deformation of the upper lip. It requires only one stage and reduces the risk of venous congestion or malnutrition ( Fig. 6 ). This procedure is not suitable for men with beard on the entire skin of the upper lip.
